# Role of hsa_circ_0000280 in regulating vascular smooth muscle cell function and attenuating neointimal hyperplasia via ELAVL1

**DOI:** 10.1007/s00018-022-04602-w

**Published:** 2022-12-07

**Authors:** Zunzhe Wang, Huating Wang, Chenghu Guo, Fangpu Yu, Ya Zhang, Lei Qiao, Haijun Zhang, Cheng Zhang

**Affiliations:** 1 The Key Laboratory of Cardiovascular Remodeling and Function Research, Chinese Ministry of Education, Chinese National Health Commission and Chinese Academy of Medical Sciences, State and Shandong Province Joint Key Laboratory of Translational Cardiovascular Medicine, Department of Cardiology, Qilu Hospital, Cheeloo College of Medicine, Shandong University, 107 Wenhuaxi Road, Jinan City, 250012 Shandong China; 2 Department of Geriatric Cardiology, Shandong Provincial Hospital Affiliated to Shandong First Medical University, Jinan, 250021 Shandong China; 3 Department of Cardiology, Jinan Central Hospital Affiliated to Shandong First Medical University, Jinan, 250013 Shandong China; 4 Institute of Vascular Intervention, Medical College of Tongji University, Shanghai, 200072 China

**Keywords:** Circular RNA, RNA-binding protein, Neointimal hyperplasia, Atherosclerosis, Coronary heart disease

## Abstract

The pathological proliferation of cells in vascular smooth muscle underlies neointimal hyperplasia (NIH) development during atherosclerosis. Circular RNAs (circRNAs), which represent novel functional biomarkers and RNA-binding proteins, contribute to multiple cardiovascular diseases; however, their roles in regulating the vascular smooth muscle cell cycle remain unknown. Thus, we aimed to identify the roles of circRNAs in vascular smooth muscle during coronary heart disease (CHD). Through circRNA sequencing of CHD samples and human antigen R (ELAVL1) immunoprecipitation, we identified circRNAs that are associated with CHD and interact with ELAVL1. Our results suggested that the hsa_circ_0000280 associated with CHD inhibits cell proliferation and induces ELAVL1-dependent cell cycle arrest. Gain/loss-of-function experiments and assays in vivo indicated that hsa_circ_0000280 facilitates interactions between ELAVL1 and cyclin-dependent kinase suppressor 1 (*CDKN1A*) mRNA and stabilization of this complex and leads to cell cycle arrest at the G1/S checkpoint, inhibiting cell proliferation of vascular smooth muscle cells in vitro and NIH in vivo. Importantly, hsa_circ_0000280 reduced neointimal thickness and smooth muscle cell proliferation in vivo. Taken together, these findings reveal a novel pathway in which hsa_circ_0000280 facilitates the regulation of ELAVL1 on *CDKN1A* mRNA to inhibit NIH. Therefore, measuring and modulating their expression might represent a potential diagnostic or therapeutic strategy for CHD.

**Supplementary Information:**

The online version contains supplementary material available at 10.1007/s00018-022-04602-w.

## Introduction

Neointimal hyperplasia (NIH) is defined as the migration and proliferation of smooth muscle cells in the tunica intima, leading to thickened arterial walls and reduced luminal space. NIH plays an integral role in the development of coronary heart disease (CHD) [1]. A crucial feature of intimal hyperplasia is the activation, migration, and subsequent multiplication of vascular smooth muscle cells (VSMCs) [2–4] that are the main cellular targets in neointimal lesions for preventing of NIH.

Circular ribonucleic acids (circRNAs) are highly conserved, long non-coding RNAs (lncRNAs) that can form stable closed loops lacking 5ʹ-end caps and 3ʹ-end poly (A) tails [5]. Unlike linear RNAs, circRNAs form covalent ring structures without 3ʹ and 5ʹ ends, rendering them relatively impervious to exonuclease degradation, and thus more stable than linear RNAs. Circular RNAs are composed primarily of exons with some intronic circularization [6]. Some circRNAs are loaded with microRNA (miRNA) response elements (MREs) that can compete with endogenous RNAs (ceRNAs), by binding to miRNAs and inhibiting their target gene expression. Moreover, circRNAs can alter protein functions either by binding directly to the protein or indirectly through RNA mediation. Indeed, studies of circRNAs in cardiovascular diseases [7–9] have included the relationship between circRNAs and the cell cycle. Specifically, circ-FOXO3 suppresses cell cycle progression by binding to the cell cycle proteins, cyclin-dependent kinase 2 (CDK2), and cyclin-dependent kinase suppressor 1 (CDKN1A, p21) to create a ternary complex [10]. However, in atherosclerotic NIH, the mechanism through which circRNAs regulate the SMC cell cycle remains unclear.

Human antigen R (ELAVL1, HuR), a member of the embryonic lethal abnormal vision (ELAV)/Hu family, is an RNA-binding protein that regulates RNA metabolism and is involved in neuronal development, proliferation, and migration. It binds U-rich sequences or AU-rich elements (ARE) in the 3ʹ-untranslated regions (UTRs) of target mRNAs [11–13]. Moreover, increased cytoplasmic expression of ELAVL1 is related to hyperplasia of the intimal and neointimal layers [14]. Many lncRNA functions are influenced by ELAVL1 binding.

Additionally, circAGO2, derived from the Argonaute 2 (*AGO2*) gene, is part of a miRNA-induced silencing complex involved in tumor progression that binds and activates the ELAVL1 protein [15]. Meanwhile, elevated levels of lncRNA OIP5-AS1 increase the formation of ELAVL1-OIP5-AS1, which hinders interactions between ELAVL1 and target mRNAs, including those encoding proteins involved in proliferation [16]. Furthermore, lncRNAs derived from the MIR100 host gene interact with ELAVL1 and several ELAVL1-target mRNAs, resulting in cell cycle regulation [17]. However, little is known about the roles of circRNAs and ELAVL1 in SMC proliferation and cell cycle regulation.

To identify key CHD-related circRNAs that interact with ELAVL1, we combined sequenced circRNAs isolated from CHD samples with those that co-precipitated with ELAVL1, then studied their functions in vitro and in vivo. The functional circRNAs associated with CHD identified in this study might serve as biomarkers and/or therapeutic targets.

## Methods

### Human arterial tissues and peripheral blood samples

Blood samples were obtained from 70 patients with CHD and 30 control patients from the Department of Cardiology at Qilu Hospital, Shandong University, China after providing written, informed consent to participate in this study. We then selected controls and patients with CHD based on the results of diagnostic coronary angiography. Patients with lung, liver, kidney, immune, or other heart diseases were excluded. Table S1 shows details of the patients. All participants received a conventional dose of aspirin and clopidogrel before percutaneous coronary intervention (PCI). Patients with > 50% and < 50% coronary stenosis were respectively assigned to the CHD and control groups [18]. Peripheral blood mononuclear cells (PBMCs) were processed, dispersed using separation medium (Solarbio, Beijing, China) and centrifuged twice at 2,000 rpm for 40 min. The PBMCs were placed in TRIzol LS (Invitrogen, Carlsbad, CA, USA) and stored at − 80 °C.

We acquired coronary artery samples from five donors with brain death (DBD) at the Organ Transplantation and Donation Department at Qilu Hospital. Family members provided written informed consent post-mortem. Samples were stored in cold HypoThermosol FRS Preservation Solution (Stemcell Technology, Bothell, WA, USA) at 4 °C within 30 min. The coronary arteries were immediately dissected using a stereomicroscope into 5–6 mm arterial rings with or without plaques, and placed in liquid nitrogen for long-term storage.

The present study proceeded according to the principles enshrined in the Declaration of Helsinki (2013 amendment). The Medical Institutional Ethics Committee of Qilu Hospital approved the collection and use of human blood and vessel samples (Prot. KYLL-2019-080).

### Western blotting, qPCR, and other techniques

Detailed protocols can be found in the Supplementary Tables S2 and S3 show details of the study protocols.

### Statistical analysis

All experiments comprised at least three independent replicates and groups contained five animals each. Differences between groups of continuous and data categorical were determined using Student *t* tests, and *χ*^2^ tests, respectively, and the results of multiple comparisons were assessed using one-way ANOVA. Data are presented as means ± SD unless otherwise indicated. All data were analyzed using SPSS 25.0 (IBM Corp., Armonk, NY, USA) or GraphPad Prism 8 (GraphPad Software, La Jolla, CA, USA). Values with *p < *0.05 were considered statistically significant.

## Results

### Expression of hsa_circ_0000280 is downregulated in coronary heart disease

**Fig. 1:**
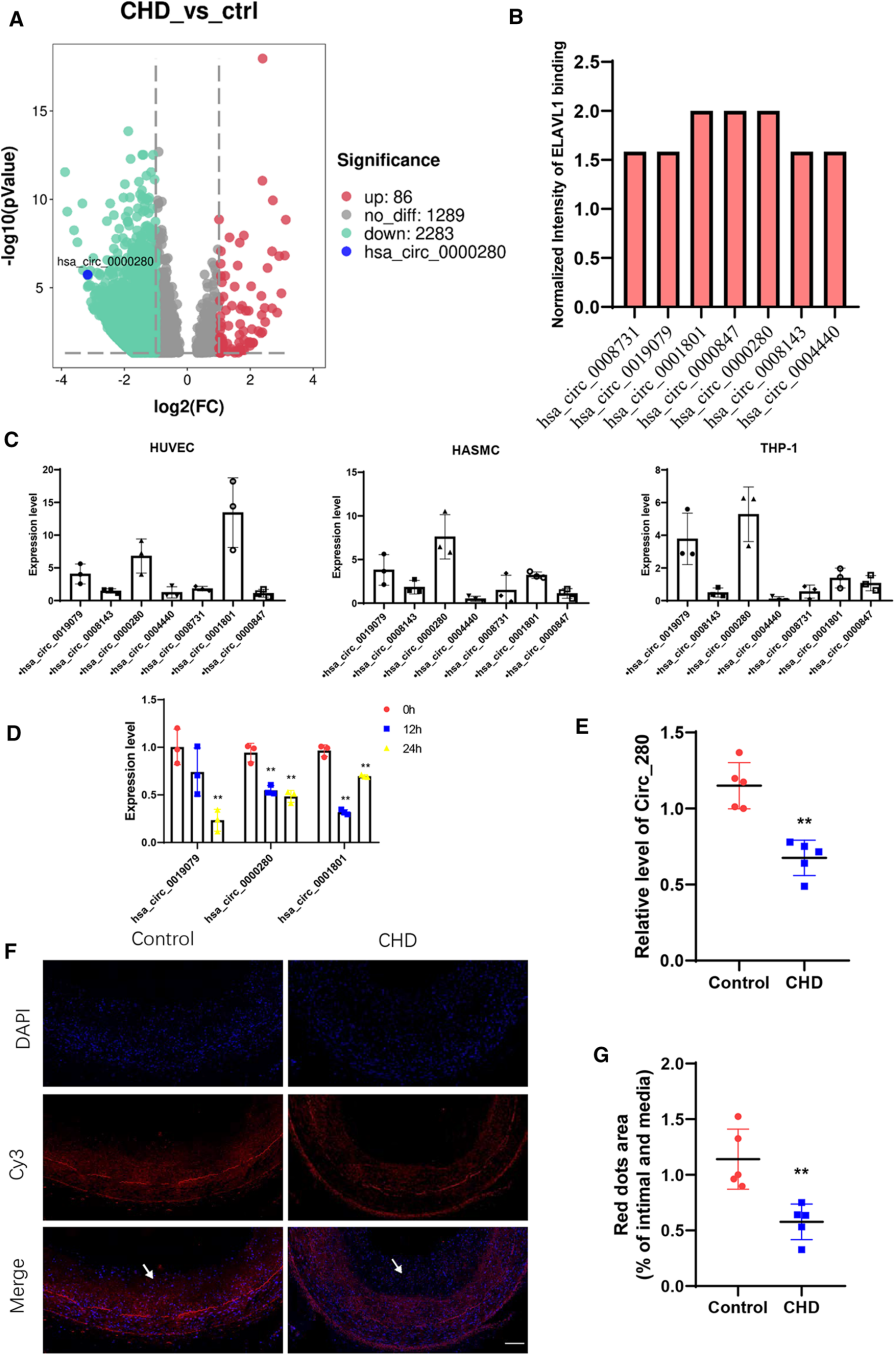
Secondary sequencing uncovered CHD-related PBMC circRNAs, validated by qRT-PCR in human atherosclerotic vessels. **A** Volcano plot of sequenced PBMC circRNAs. X-axis, fold modification expressed as log2; Y-axis: *p* value expressed as − log10. Horizontal line represents *p = *0.05. Compared with the control, red and green points exhibited significantly upregulated and downregulated circRNAs at *p < *0.05 and *p < *0.05, respectively. Blue point represents hsa_circ_0000280 (CHD, *n = *70; control, *n = *30; Student *t* test). **B** ELAVL1 RIP-sequencing revealed top seven circRNAs. **C** Abundance of top seven circRNAs in three types of cardiovascular cells (*n = *3). **D** Expression of three circRNAs measured in PDGF-BB-induced HASMCs (*n = *3, *p < *0.01; Student *t* tests). **E** Quantitative RT-PCR analysis of hsa_circ_0000280 in human coronary arteries with and without (matched controls) luminal stenosis. (*n = *5 pairs, *p = *0.005; Student *t* test). **F**, **G** Fluorescence in situ hybridization assay (FISH) shows location and expression of hsa_circ_0000280 in control and CHD tissues. White arrows indicate intimal layer. Probes for hsa_circ_0000280 were labeled with Cy3. Scale bar, 100 µm (*n = *5 pairs, *p = *0.0038; Student *t* tests). Data are presented as means ± SD

We sequenced 30 non-CHD and 70 CHD samples of PBMCs from the patients to identify potential circRNA biomarkers associated with CHD. Compared with controls, 86 circRNAs were significantly upregulated; whereas 2,283 were downregulated, including hsa_circ_0000280 (Fig. 1A, Supplementary Table S4). We combined downregulated circRNA and ELAVL1 RIP-circRNA sequencing to filter out targeted circRNAs that were associated with CHD and had strong ELAVL1 binding activity (Fig. 1B, Supplementary Table S5). Supplementary Table S6 shows the top seven circRNAs. We then determined the levels of these circRNAs in three human vascular cell types (Fig. 1C) and found that hsa_circ_0000280 was enriched in human aortic smooth muscle cells (HASMCs). The platelet-derived, dimeric growth factor (PDGF)-BB-induced HASMC model has been accepted for studies of cell proliferation in vascular smooth muscle and migration [19, 20]. Therefore, we analyzed the expression of circRNAs in HASMCs induced with PDGF-BB and found that three, including hsa_circ_0000280, were downregulated (Fig. 1D). We then investigated the expression of hsa_circ_0000280 in five matched human coronary artery samples by qPCR. Compared with without plaque, the abundance of hsa_circ_0000280 was significantly decreased in coronary arteries that contained plaques compared with those that did not (Fig. 1E). We verified the presence and distribution of hsa_circ_0000280 in human vessels using a probe for the junction point of hsa_circ_0000280 that we designed and fluorescent in situ hybridization (FISH). The results revealed hsa_circ_0000280 in human coronary vessels, mostly in the tunica media. However, compared with the control, the hsa_circ_0000280 signal was weaker in the CHD tissue (Fig. 1F and G). Taken together, these results suggested an association between hsa_circ_0000280 and CHD, and its potential to serve as a CHD biomarker.

### Identification of hsa_circ_0000280

**Fig. 2:**
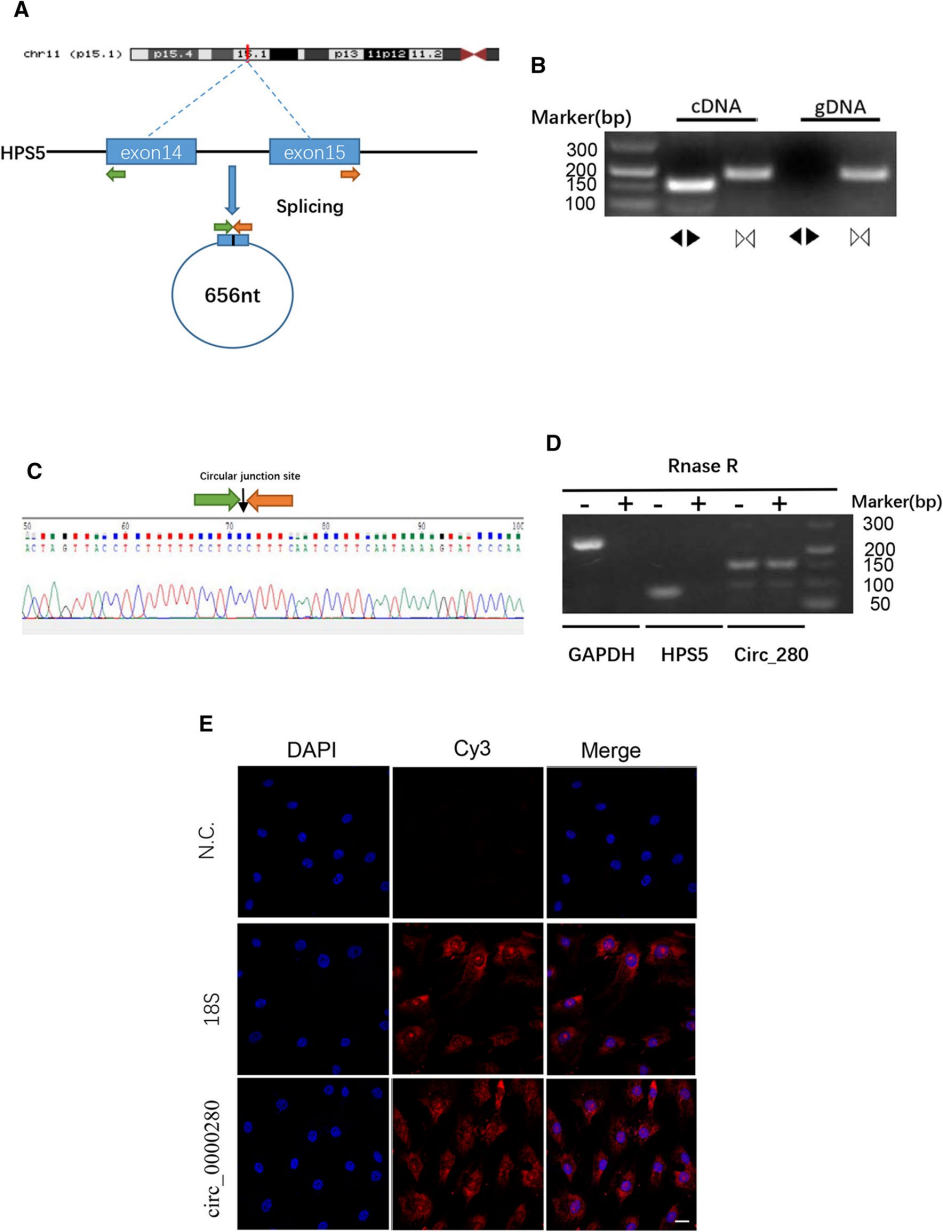
Specification and location of hsa_circ_0000280. **A** Scheme of hsa_circ_0000280 production. **B** Specific divergent and convergent primers amplified hsa_circ_0000280 in cDNA and genomic DNA (gDNA) from HASMCs. **C** Circular junction site of hsa_circ_0000280 revealed by sequencing. **D** Validation of hsa_circ_0000280 Stability using RNase R and RT-PCR analysis (*n = *3, *p < *0.05; Student *t* test). **E** Fluorescence in situ hybridization assay (FISH) shows hsa_circ_0000280 localization in HASMCs. Control was18s rRNA. Probes for hsa_circ_0000280 and 18 s rRNA were labeled with Cy3. Scale bar, 20 µm. Data are presented as means ± SD

We located hsa_circ_0000280 on chromosome 11: 18,312,988–18,314,523 in the human genome, derived from the exon-14 and exon-15 circularization procedures of the *HPS5* gene. The length of hsa_circ_0000280 was found to be 656 nt (Fig. 2A). To confirm the circularization of hsa_circ_0000280, we designed specific hsa_circ_0000280 convergent and divergent primers in cDNA and gDNA. Convergent primers were amplified in RNA-derived and genomic DNA specimens. However, divergent primers created amplicons from RNA-derived specimens but not from genomic DNA, which confirmed the specificity of the divergent primers (Fig. 2B). Moreover, Sanger DNA sequencing confirmed head-to-tail splicing in the HASMC sample (Fig. 2C). Ribonuclease R preferentially degrades linear RNA sequences [21, 22]. Thus, we digested RNA with this enzyme and quantified the resistance of hsa_circ_0000280 together with the other two linear RNAs using qPCR. The results showed that hsa_circ_0000280 was more resistant to digestion than the linear control mRNAs, Biogenesis of Lysosomal Organelles Complex 2 Subunit 2 (*HPS5*) and glyceraldehyde-3-phosphate dehydrogenase (*GAPDH*) (Fig. 2D). We found using FISH that hsa_circ_0000280 in HASMCs localized predominantly in the cytoplasm (Fig. 2E), allowing them to function as miRNA or protein sponges.

### hsa_circ_0000280 interacts with ELAVL1

**Fig. 3:**
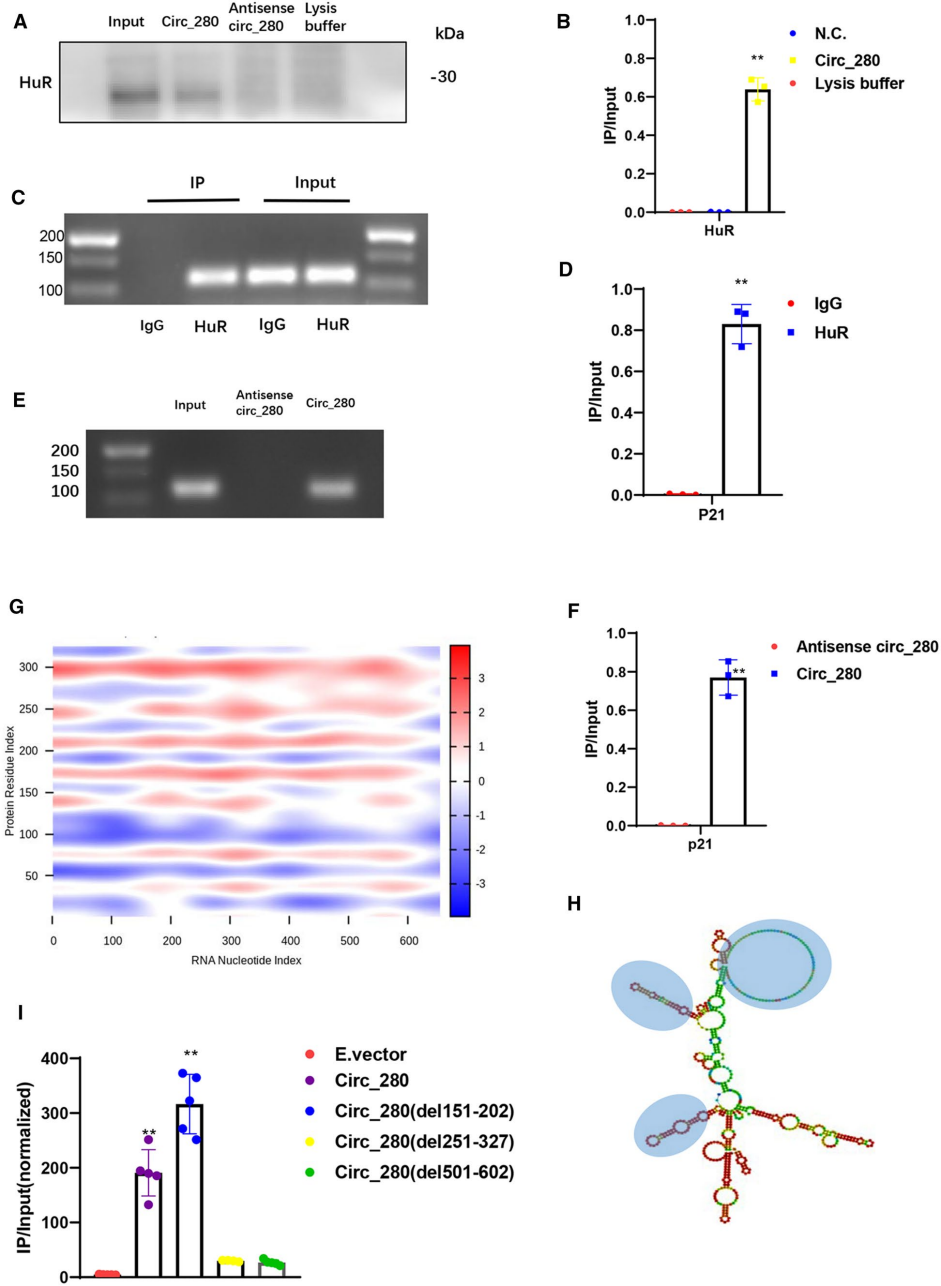
Hsa_circ_0000280 binds ELAVL1. **A**, **B** RNA pull-down assay of hsa_circ_0000280 in vitro. Negative controls comprised antisense RNA of hsa_circ_0000280 and blank lysis buffer. Western blots of ELAVL1 protein (*n = *3, *p < *0.0001; Student *t* tests). **C**, **D** RNA immunoprecipitation of ELAVL1 in HASMCs. CDKN1A RT-PCR harvest determined by agarose gel electrophoresis, and relative immunoprecipitated (IP)/input ratios are shown (*n = *3, *p < *0.0001; Student *t* test). **E**, **F** RNA pull-down assay using hsa_circ_0000280 probe in vitro. Antisense RNA of hsa_circ_0000280 was negative control (NC). Harvested CDKN1A RT-PCR was determined by agarose gel electrophoresis, and relative immunoprecipitated (IP)/input ratios are shown (*n = *3, *p < *0.0001; Student *t* tests). **G** Prediction of RNA–protein interaction between hsa_circ_0000280 with ELAVL1 using catRAPID algorithm. **H** Secondary structure prediction of hsa_circ_0000280 using Vienna RNA Web Services. The blue circle shows predicted top three binding sites of hsa_circ_0000280 for ELAVL1. **I** RIP of ELAVL1 from empty vector (E.vector), circ_280, and three circ-deleted groups. Bound hsa_circ_0000280 was determined by qPCR (*n = *5, *p < *0.01; Student *t* tests). Data are presented as means ± SD

We reconfirmed the RIP-sequencing results using RIP-agarose gel electrophoresis and RNA pull-down-western blotting in HASMCs (Supplementary Fig. S1A and B). When hsa_circ_0000280 was pulled down, ELAVL1 protein was specifically enriched but the antisense probe did not bind to it (Fig. 3A and B). As an established RNA stabilizer protein, ELAVL1 is an established stabilizes several mRNAs involved in cell cycle progression, such as CDKN1A, CCND, CCNE, and CDK2[17, 23–27]. We, therefore, confirmed the interaction of ELAVL1 protein with *CDKN1A* mRNA via RIP (Fig. 3C, D). We then performed an RNA pull-down assay and observed the interaction between hsa_circ_0000280 and CDKN1A mRNA (Fig. 3E, F). FISH of ELAVL1, hsa_circ_0000280, and *CDKN1A* mRNA confirmed their binding (Fig. S2). These results suggest that hsa_circ_0000280 can bind to ELAVL1 protein and *CDKN1A* mRNA, providing potential evidence for the function study of hsa_circ_0000280.

Furthermore, using the catRAPID and Vienna RNA algorithm for RNA–protein interactions and circRNA secondary structure analyses, the binding of hsa_circ_0000280-ELAVL1 was predicted to occur between three major RNA regions (151–202, 251–327, and 501–602 nucleotides), and five ELAVL1 protein domains (Fig. 3G, H). The five domains in ELAVL1 are highly homologous between humans and mice. Therefore, to determine if the three predicted regions of hsa_circ_0000280 were necessary for ELAVL1 binding, we designed overexpression plasmids with deleted ELAVL1 binding sites. The RIP-qPCR results showed that the ELAVL1 antibody precipitation ratio of the hsa_circ_0000280(del151-202) group was consistent with the ratio for the E.vector and WT hsa_circ_0000280 groups, whereas that of hsa_circ_0000280(del251-327) and hsa_circ_0000280(del501-602) was decreased (Fig. 3I). These findings indicate that the RNA regions 251–327 and 501–602, and not 151–202, are likely indispensable for the interaction between hsa_circ_0000280 and ELAVL1.

### Functions of HASMCs are regulated by hsa_circ_0000280

**Fig. 4:**
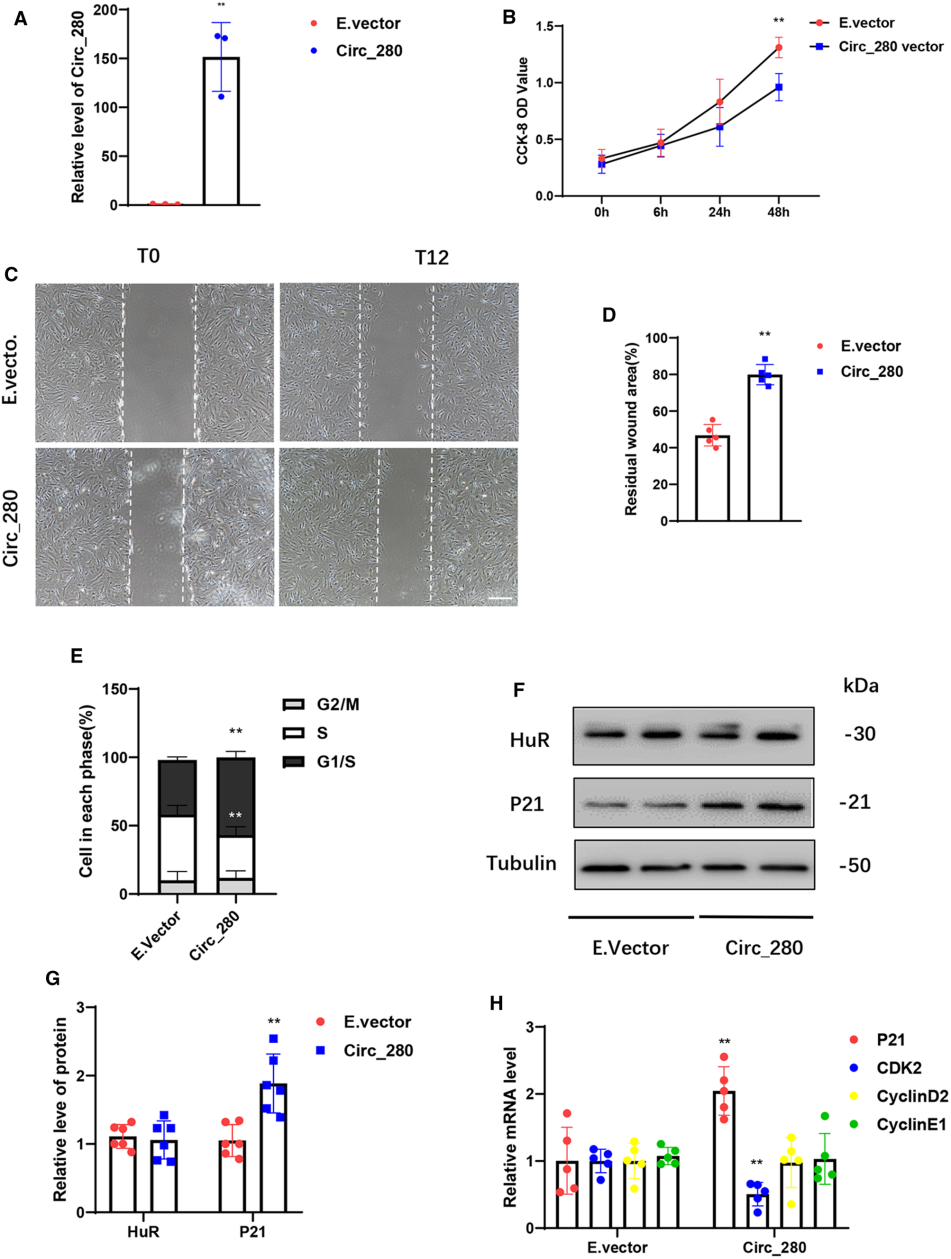
Biological effects of hsa_circ_0000280 determined by gain-of-function assays in HASMCs. **A** Expression of hsa_circ_0000280 elevated by plasmid overexpression (*n = *3, *p = *0.0018; Student *t* test). **B** HASMC cell proliferation determined using CCK8 assays. X-axis: duration of hsa_circ_0000280 stimulation by plasmid (*n = *3, compared with 0 h, *p = *0.0058; Student *t* tests). **C**, **D** Wound assays of hsa_circ_0000280-elevated HASMCs. Graph shows quantified residual wounded area at 12 h post-scratch *vs*. empty vector (E.vector) controls. (*n = *5, *p < *0.0001; Student *t* tests) Scale bar, 100 μm. **E** Cell cycle assays of HASMCs incubated with exogenous hsa_circ_0000280 *vs*. E.vector controls (*n = *3, *p < *0.05; Student *t* tests). **F**, **G** Western blots of ELAVL1 and CDKN1A in hsa_circ_0000280-elevated HASMCs *vs.* E.vector controls (*n = *6, *p = *0.0019; Student *t* tests). **H** Expression of CDKN1A, CDK2, CCND2, and CCNE1 determined by qPCR in hsa_circ_0000280-elevated HASMCs *vs*. E.vector controls (*n = *5, *p < *0.01; Student *t* tests). Data are presented as means ± SD

We also evaluated the role of hsa_circ_0000280 in HASMC homeostasis using loss- and gain-of-function studies. We generated an overexpression plasmid that substantially increased hsa_circ_0000280 expression (Fig. 4A). Exogenously overexpressed hsa_circ_0000280 decreased HASMC proliferation by 36.42% (Fig. 4B). The results of wound-healing assays indicated a significant reduction in the HASMC migratory capacity when exogenous hsa_circ_0000280 expression was induced (Fig. 4C and D). Cell cycle analysis further revealed that exogenous hsa_circ_0000280 diminished the proportion of HASMCs in the S phase and induced their accumulation in the G1/S phase (Fig. 4E). We then investigated the influence of hsa_circ_0000280 on ELAVL1 and ELAVL1-target genes. We found that increasing the abundance of hsa_circ_0000280 did not alter ELAVL1 protein levels, but enhanced those of CDKN1A (Fig. 4F and G). Exogenous hsa_circ_0000280 did not affect CCND2 or CCNE1 expression but inhibited that of CDK2 (Fig. 4H).

We transfected siRNA to decrease hsa_circ_0000280 levels (Supplementary Fig. S3A). Downregulated endogenous hsa_circ_0000280 promoted HASMC proliferation by 55.1% (Fig. S3B) and increased their migratory capacity (Supplementary Fig. S3C and D). Cell cycle analysis further revealed that decreased hsa_circ_0000280 expression diminished HASMC accumulation at the G1/S phase with more cells remaining in the S phase (Fig. S3E). Reduced hsa_circ_0000280 also decreased mRNA levels of *CDKN1A*, but not ELAVL1 (Fig. S3F). Moreover, knocking down hsa_circ_0000280 decreased the expression of VSMC differentiation markers, including actin and transgelin, in differentiated HASMCs that have less migratory and proliferative capacity [28, 29] (Supplementary Fig. S3F). However, CDK2, CCND2, and CCNE1 expression was not affected by si_circ_280 transfection (Supplementary Fig. S3G). To exclude the possibility that hsa_circ_0000280 exerts its effect through altering HPS5 expression, we quantified its expression and found that HPS5 expression did not significantly differ in cells transfected with hsa_circ_0000280 siRNAs or plasmids (Supplementary Fig. S3H and I). This excluded the possibility that hsa_circ_0000280 exerts its effects by altering HPS5 expression.

### Effects of hsa_circ_0000280 on HASMCs depend on ELAVL1 and CDKN1A

**Fig. 5:**
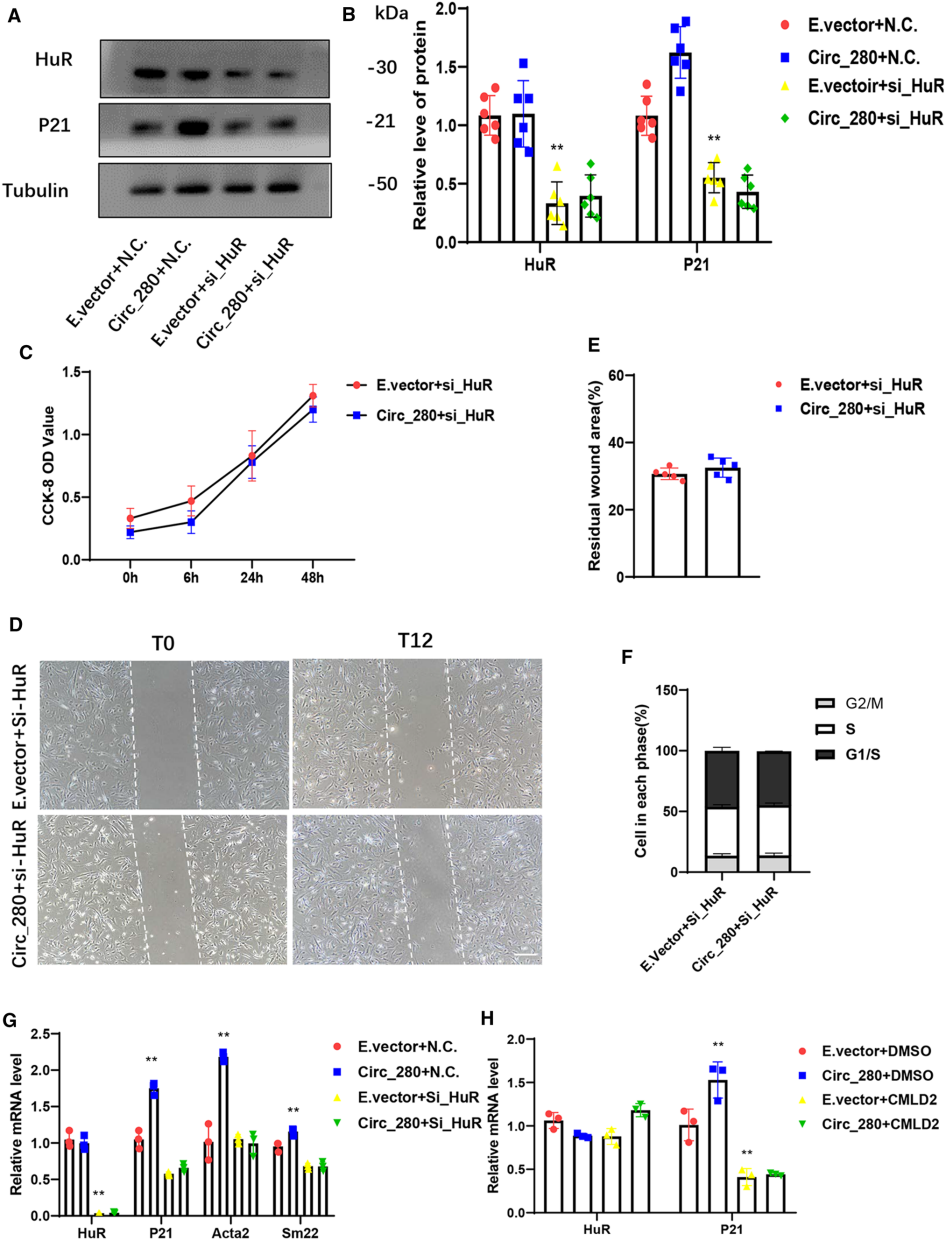
Inhibitory effect of hsa_circ_0000280 on cell proliferation depends on ELAVL1. **A** ELAVL1 and hsa_circ_0000280 were modified by hsa_circ_0000280 plasmid and ELAVL1 siRNA. **B** Western blots of ELAVL1 and CDKN1A protein levels (*n = *6) compared with E.vector + NC, *p < *0.001; compared with E.vector + si_ELAVL1, *p > *0.05; multiple *t* tests). **C** HASMC cell proliferation determined by CCK8 assay. X-axis, duration of hsa_circ_0000280 stimulation by plasmid (*n = *6) compared with 0 h, *p > *0.05; Student *t* tests). **D**, **E** Wound assay of hsa_circ_0000280-elevated HASMCs after si_ELAVL1 transfection. Graph shows quantitation of residual wounded area at 12 h post-scratch *vs.* E.vector controls (*n = *5, *p > *0.05; Student *t* tests). Scale bar, 100 μm. **F** Cell cycle analysis of hsa_circ_0000280-elevated HASMCs *vs*. E.vector controls (*n = *3, *p > *0.05; Student *t* tests). **G** Expression of actin (ACTA2) and transgelin (TAGLN1, Sm22) in has_circ_0000280 plasmid and si_ELAVL1 *vs.* control (E.vector and NC). VSMCs, assessed by qPCR (*n = *3; *p < *0.05 and *p > *0.05 compared with E.vector + NC and E.vector + si_ELAVL1, respectively; multiple *t* tests). **H** Addition of ELAVL1 inhibitor, CMLD-2. Levels *ELAVL1* and *CDKN1A* mRNA were quantified by qPCR. Control was DMSO for CMLD-2 (*n = *3; *p < *0.01 and *p > *0.05 compared with E.vector + DMSO and E.vector + CMLD-2, respectively; multiple *t* tests). Data are presented as means ± SD

We examined the biological effects of hsa_circ_0000280 in HASMCs without ELAVL1 to confirm the above findings. We designed an ELAVL1 siRNA and transfected it before the addition of exogenous hsa_circ_0000280. ELAVL1 levels were inhibited after si-ELAVL1 transfection. Exogenous hsa_circ_0000280 did not alter CDKN1A expression after si-ELAVL1 infection (Fig. 5A and B). ELAVL1 downregulation also negated the influence of exogenous hsa_circ_0000280 on cell proliferation (Fig. 5C). Wound-healing assays further indicated that increasing hsa_circ_0000280 did not affect a residual wound area in the absence of ELAVL1 (Fig. 5D and E). Similarly, exogenous hsa_circ_0000280 did not affect cell cycle progression after silencing ELAVL1 (Fig. 5F). Elevated hsa_circ_0000280 levels promoted ACTA2 and TAGLN1 expression. However, ELAVL1 inhibition also abolished the effects of exogenous hsa_circ_0000280 on VSMC differentiation markers (Fig. 5G). To further verify these findings, we added the specific ELAVL1 inhibitor, chloromuconolactone dehalogenase (CMLD-2), and the results were similar to those of siRNA (Fig. 5H). Taken together, these data suggest that hsa_circ_0000280 participates in the proliferation, migration, and differentiation of HASMCs, and that ELAVL1 serves as an essential factor in these functions.

**Fig. 6:**
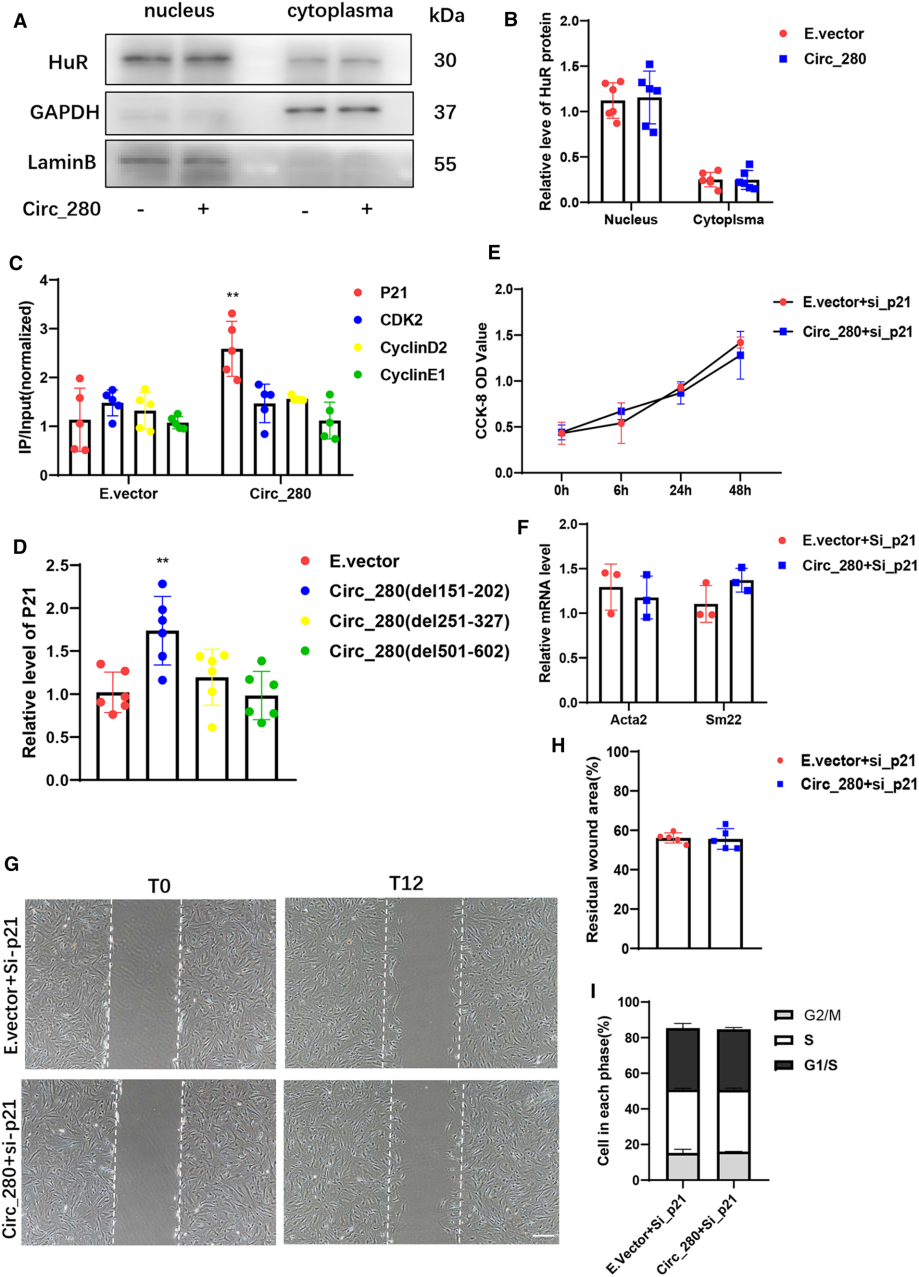
Effects of hsa_circ_0000280 are dependent on CDKN1A. **A**, **B** Western blots of ELAVL1 relative nuclear and cytoplasmic abundance in E.vector and Circ_280 groups. Results are shown as ratios (*n = *6; *p > *0.05; Student *t* tests). **C** RNA immunoprecipitation of ELAVL1 in HASMCs after exogenous hsa_circ_0000280 delivery *vs*. E.vector (*n = *5; *p = *0.0053; Student *t* tests). **D** Relative *CDKN1A* mRNA level after transfection with hsa_circ_0000280 harboring various deletions (*n = *6; *p = *0.0035 compared with E.vector; Student *t* tests). **E** Proliferation of HASMCs with *CDKN1A* knock down determined by CCK8 assays. X-axis, duration of stimulation by hsa_circ_0000280 plasmid. (*n = *6, compared with 0 h, *p > *0.05; Student *t* tests). **F** Expression of actin (ACTA2) and transgelin (Sm22) in hsa_circ_0000280 plasmid and si_CDKN1A *vs.* control (E.vector + si_CDKN1A; *n = *3, *p > *0.05; Student *t* tests). **G**, **H** Wound assays of hsa_circ_0000280-elevated HASMCs transfected with si_CDKN1A. Graph shows quantitation of residual wounded area at12 h post-scratch *vs.* E.vector controls (*n = *5, *p > *0.05; Student *t* tests). Scale bar, 100 μm. **I** Cell cycle analysis of hsa_circ_0000280-elevated HASMCs *vs*. E.vector controls (*n = *3, *p > *0.05; Student *t* test). Data are presented as means ± SD

The function of ELAVL1 is closely associated with its subcellular distribution [13, 17, 24]. We detected ELAVL1 in the cytoplasm and nucleus but did not observe a significant change in ELAVL1 subcellular localization after addition of exogenous hsa_circ_0000280 (Fig. 6A and B). The RIP-RT-qPCR result indicated that ELAVL1 had a greater interaction with cyclin-dependent kinase inhibitor 1A (*CDKN1A*) than with other target mRNAs after hsa_circ_0000280 overexpression (Fig. 6C). Moreover, the results of deletion experiments showed that *CDKN1A* mRNA upregulation was abolished by circ_280(del251-327) and circ_280(del501-602), which were similarly expressed (Figs. 6D, S4). These outcomes indicated that the association between ELAVL1 and *CDKN1A* mRNA detected upon hsa_circ_0000280 overexpression is not due to ELAVL1 abundance or localization, but rather to changes in its ability to interact with *CDKN1A* mRNA.

To confirm the function of CDKN1A in hsa_circ_0000280-induced biological effects, we knocked down *CDKN1A* with an siRNA and transfected it along with exogenous hsa_circ_0000280 (Supplementary Fig. S5). Downregulated CDKN1A resulted in suppressing the influence of exogenous hsa_circ_0000280 on cell proliferation (Fig. 6E) and its effects on VSMC differentiation markers (Fig. 6F). Wound-healing assays indicated that exogenous hsa_circ_0000280 did not affect the residual wound area after CDKN1A depletion (Fig. 6G and H). The results were similar observed after silencing *ELAVL1*, and upregulated hsa_circ_0000280 did not affect cell cycle progression after silencing CDKN1A (Fig. 6I). These results suggested that the biological effects of hsa_circ_0000280 depend on both ELAVL1 and CDKN1A.

### Neointimal hyperplasia in vivo is reduced by hsa_circ_0000280

**Fig. 7:**
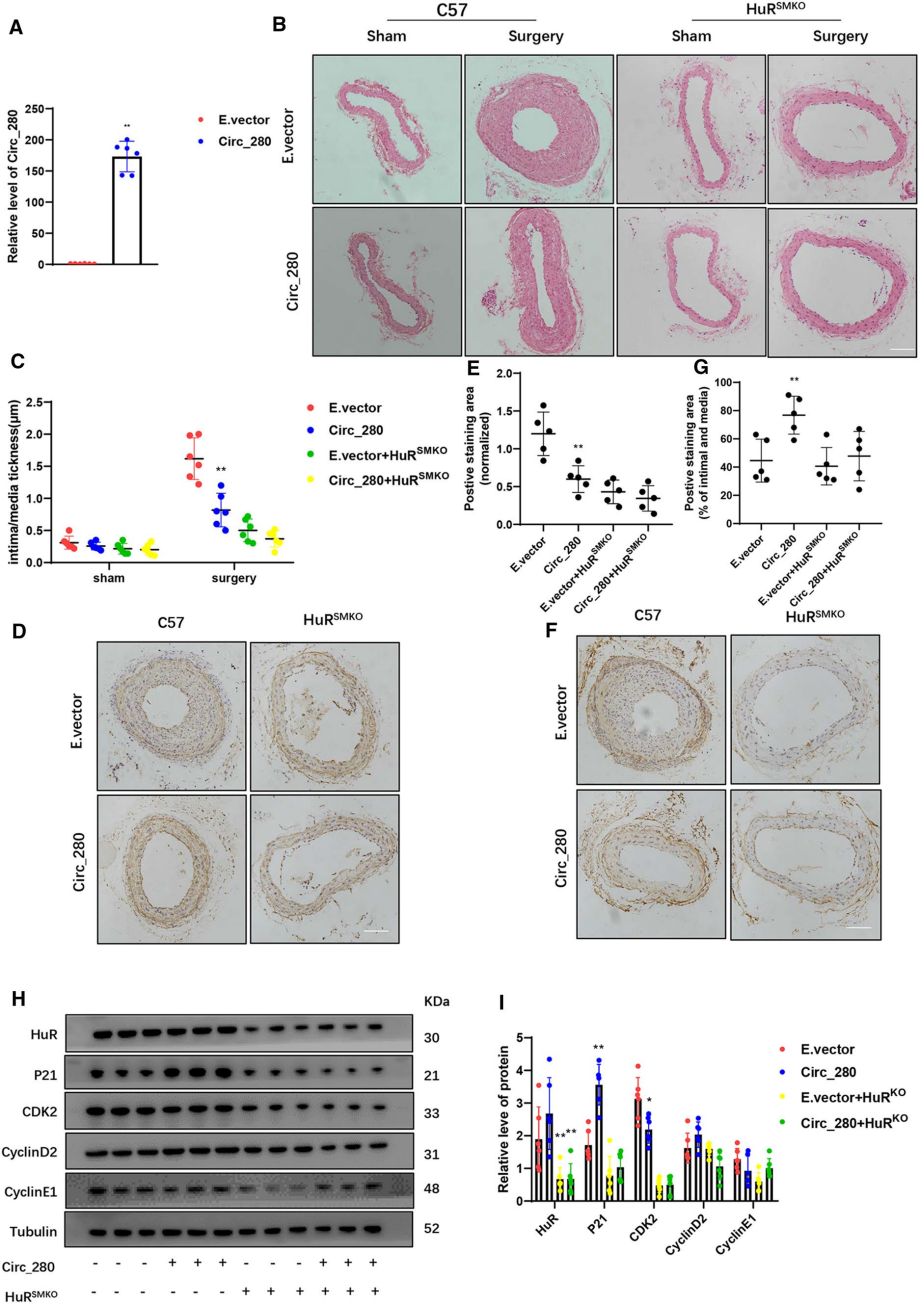
Neointimal hyperplasia is reduced by hsa_circ_0000280 in vivo. **A** Delivery of hsa_circ_0000280 into CCA completed. Level of hsa_circ_0000280 detected by qPCR (*n = *6; *p < *0.0001; Student *t* tests). **B** Hematoxylin–Eosin staining of intimal changes induced by ligation surgery after hsa_circ_0000280 delivery *vs.* E.vector. **C** Intimal/media thickness measurements (Scale bar, 100 µm; *n = *6; *p = *0.0008; two-way ANOVA, multiple *t* tests). **D–G** Representative immunohistochemical staining of SMA and CDKN1A (Scale bar, 100 µm; *n = *5; *p = *0.0444 vs. E.vector; *p = *0.0079 compared with E.vector + ELAVL1^SMKO^; *p > *0.05; one-way ANOVA, multiple *t* tests). **H**, **I** Western blots of indicated proteins in CCA of four groups of mice (*n = *6 per group; *p < *0.05 vs. E.vector; *p > *0.05 vs. E.vector + ELAVL1^SMKO^; two-way ANOVA, multiple *t* tests). Data are presented as means ± SD

We determined whether the modulation of hsa_circ_0000280 impacts the pathological response after vascular injury in murine models. We ligated the unilateral CCA at 21 days in WT and ELAVL1^SMKO^ mice, which comprise an established model of neointimal hyperplasia [30–32]. Hsa_circ_0000280 was delivered to the CCA using an SMC-specific adeno-associated virus AAV9-SM22a-Circ_280, and delivery was confirmed by qPCR (Fig. 7A).

We found that while ligation of the CCA led to strong intimal hyperplasia, overexpressed hsa_circ_0000280 decreased the neointimal area stained with hematoxylin and eosin (HE). Hsa_circ_0000280 did not affect neointimal areas in ELAVL1^SMKO^ mice (Fig. 7A and B). To measure the area of SMCs in each section, immunostaining for α-SMA was performed. This revealed fewer positive cells in the AAV9-SM22a-Circ_280 group than in the E.vector group, and that the numbers of α-SMA positive cells did not significantly differ in ELAVL1^SMKO^ mice treated with E.vector or AAV9-SM22a-Circ_280 (Fig. 7D and E). Immunostaining for CDKN1A protein showed stronger staining in the AAV9-SM22a-Circ_280 group than in the E.vector group, with no differences in positive cells in ELAVL1^SMKO^ mice (Fig. 7F and G). Protein levels did not change in the presence of hsa_circ_0000280, ELAVL1, CCND2, or CCNE1. However, CDKN1A protein levels increased when CDK2 was inhibited in the AAV9-SM22a-Circ_280 group. Both proteins did not significantly differ in ELAVL1^SMKO^ mice treated with E.vector or AAV9-SM22a-Circ_280 (Fig. 7H and I).

Overall, these findings showed that hsa_circ_0000280 regulates the biological activities of SMCs in vascular pathologies and that the effect of hsa_circ_0000280 on vascular diseases is dependent on ELAVL1.

## Discussion

As emphasis on circRNAs associated with cardiovascular diseases has increased, several circRNAs have been identified as important factors in vascular biology [7]. In VSMCs, cirANRIL (circular antisense non-coding RNA in the INK4 locus), which is transcribed at a locus of atherosclerotic cardiovascular disease on chromosome 9p21, confers vascular protection by monitoring ribosomal RNA (rRNA) maturation [33]. Furthermore, circ_Lrp6, which acts as a sponge to regulate the function of miRNA-145 is implicated in the proliferation, migration, and differentiation of VSMCs [29]. Furthermore, circActa2, which serves as a miRNA sponge for αSMA expression, is essential for SMC function [34]. These findings suggest novel strategies for further SMC investigation focused on circRNAs that regulate the progression of vascular disease.

Our findings revealed a novel circRNA that regulates the SMC cell cycle and NIH in atherosclerosis. The stable circRNA hsa_circ_0000280 is downregulated in PBMCs of patients with CHD and in human atherosclerotic vessels. Moreover, this circRNA can be accurately quantified using PBMCs isolated from collected blood samples, which is far less invasive than collecting coronary artery tissues. Thus, hsa_circ_0000280 has considerable potential for the diagnosis of CHD.

The protein ELAVL1 plays an essential role in several biological processes such as proliferation, cell cycle, and cancer growth. Specifically, ELAVL1 regulates cell division and checkpoint reactions via diverse mechanisms, including modulating the translation and stability of key regulators of the cell cycle, such as cyclins [17, 27, 35]. This protein also contributes to the stability of CDKN1A mRNA, which is a cyclin-dependent kinase (CDK) suppressor in the cell cycle transition from G1 to S and G2 to M phase. Moreover, p21 affects cytoskeletal organization and cell migration via regulation of the Rho pathway [36–39].

Our loss/gain-of-function studies have shown that hsa_circ_0000280 inhibits SMC proliferation and cell cycle progression, as shown by less SMCs in the S phase and G1/S phase arrest after the delivery of hsa_circ_0000280. The primary checkpoints of G1/S, which have been demonstrated to interact with ELAVL1 are CCND, CCNE, and CDK2. However, hsa_circ_0000280 delivery elicited no significant changes in levels of CCND or CCNE, whereas those of CDK2 were downregulated. The level of CDKN1A was also increased, which might have induced G1/S phase arrest [37, 38]. We further confirmed that hsa_circ_0000280 binds to either ELAVL1 protein or CDKN1A mRNA, which further facilitates the interaction between ELAVL1 and CDKN1A. Subsequently, upregulated CDKN1A inhibits CDK, causing accumulation of cells in the G1/S phase.

Most studies on ELAVL1 in SMCs and vascular pathologies have found that ELAVL1 cooperates in the regulation of VSMC homeostasis [14, 26], with augmented ELAVL1 expression and cytoplasmic enrichment within the intimal and neointimal layers [26]. Our results for ELAVL1^SMKO^ mice are consistent with these findings.

Furthermore, ELAVL1 localization also regulates the cell cycle [40, 41]. In this context, our findings showed that hsa_circ_0000280 is distributed primarily in the cytoplasm, where the ELAVL1 effects transpired. Furthermore hsa_circ_0000280 altered neither the total level of ELAVL1 nor its subcellular localization, but rather increased interaction between ELAVL1 and its specific target, CDKN1A. This suggests that hsa_circ_0000280 can preferentially guide the binding of ELAVL1 to CDKN1A mRNA instead of CCND and CDK2 mRNA. These findings are consistent with those of a previous study, which showed that MIR100HG preferentially promotes the association between ELAVL1 and its target mRNAs [17]. Thus, hsa_circ_0000280 might act as a transporter to facilitate interactions between ELAVL1 and its target mRNA via this mechanism.

The *HPS5* gene from which hsa_circ_0000280 originates has not been investigated from the viewpoint of atherosclerosis. Although hsa_circ_0000280 regulation did not impact the mRNA level of *HPS5*, whether hsa_circ_0000280 affects homeostatic progression via HPS5 is difficult to determine. A CDKN1A knock down assay in vivo in a future study would be another way to confirm the effects of hsa_circ_0000280. In addition, hsa_circ_0000280 appears to be downregulated in PBMCs from patients. The function of hsa_circ_0000280 in PBMCs is also unknown. This might be associated with atherosclerosis caused by chronic systemic inflammation. However, additional investigations are required to confirm this postulation.

In conclusion, this study revealed the roles of a novel circRNA, hsa_circ_0000280, in regulating ELAVL1 activity in SMC and NIH. This information provides scientific evidence and guidance for future studies on circRNAs as potential diagnostic biomarkers and therapeutic targets against atherosclerosis.

## Supplementary Information

Below is the link to the electronic supplementary material.

Supplementary file1 (XLSX 261 kb)

Supplementary file2 (XLSX 61 kb)

Supplementary file3 (DOCX 5594 kb)

## Data Availability

The datasets generated during the current study are available from the corresponding author upon reasonable request.

## Abbreviations

circRNAs: Circular RNAs
CHD: Coronary heart disease
ELAVL1, HuR: Human antigen R
CDKN1A: Cyclin-dependent kinase suppressor 1
NIH: Neointimal hyperplasia
VSMCs: Vascular smooth muscle cells
CDK2: Cyclin-dependent kinase 2
