# Supplementary material for: Role of hsa_circ_0000280 in regulating vascular smooth muscle cell function and attenuating neointimal hyperplasia via ELAVL1
Source: Cell Mol Life Sci. 2022 Dec 7;80(1):3. doi: 10.1007/s00018-022-04602-w (PMC9729135; doi:10.1007/s00018-022-04602-w)
Supplement: Supplementary file 3 — Supplementary file3 (DOCX 5594 kb) [file 18_2022_4602_MOESM3_ESM.docx]

***SUPPLEMENTARY MATERIALS***

**Role of hsa_circ_0000280 in regulating vascular smooth muscle cell function and attenuating neointimal hyperplasia via ELAVL1**

Zunzhe Wang^1^, Huating Wang^2^, Chenghu Guo^1^, Fangpu Yu^1^, Ya Zhang^1^, Lei Qiao^1^, Haijun Zhang^3^, Cheng Zhang^1^*

**Supplementary Methods**

**CircRNA high-throughput sequencing**

Sequencing libraries were generated from RNAs using the NEBNext® Ultra™ Directional RNA Library Prep Kit for Illumina® (NEB, Beverly, MA, USA) following the manufacturer’s instructions. Briefly, fragmented RNA samples were used for first- and second-strand cDNA synthesis with random hexamer primers. The libraries were then purified (AMPure XP system, Beckman Coulter, High Wycombe, UK) and subsequently qualified using an Agilent Bioanalyzer 2100 system (Agilent Technologies, Santa Clara, CA, USA). After cluster generation, the library preparations were sequenced on an Illumina HiSeq 2500 platform (Illumina, San Diego, CA, USA); 125-bp paired-end reads were generated. Categorical variables were tested using the χ2 test. Continuous variables were first measured via Kolmogorov–Smirnov and Shapiro–Wilk tests to verify whether the datasets were normally distributed and subsequently analyzed using either the two-tailed Student’s *t*-test or the Mann–Whitney U test.

Raw data were cleaned using the Trimmomatic program to remove reads containing adapter sequences, invalid reads (>10% unknown bases), and low-quality reads (>50% of bases with Q-value ≤ 5). The clean reads were aligned to the reference human genome (hg38) with Bowtie2 and counted by HT-seq in union mode. Untrimmed reads that mapped contiguously to the genome were excluded. The remaining reads were used for the potential identification and characterization of circRNAs through the find circ pipeline. Briefly, anchors (extracted from both ends of the reads) that aligned in the reverse orientation (head to tail) indicated a back-spliced junction such as that found in circRNAs. The resulting splicing events were filtered according to the following criteria: a GT/AG signal flanking the splice sites, unambiguous breakpoint detection, a maximum of two mismatches during extension, breakpoint located no more than two nucleotides inside an anchor, and at least two independent reads supporting a back-spliced junction. We also excluded circRNAs with two independent reads found in only one sample to reduce the false-positive rate. Differential expression between the two groups was analyzed using DESeq2. The adjusted p-value (padj) was the p-value adjusted for multiple tests using the Benjamini-Hochberg procedure to estimate the false discovery rate. CircRNAs with a padj < 0.05 and a fold-change ≥ 2.0 or ≤ 0.5 were considered differentially expressed.

**Cell culture and treatment**

All cell lines were cultured at 37 °C with 5% CO_2_. Human aortic smooth muscle cells (HASMCs) were purchased from ScienCell Research Laboratories (Carlsbad, CA, USA) and cultured in Smooth Muscle Cell Medium (ScienCell Research Laboratories) with 2% fetal bovine serum (FBS; ScienCell Research Laboratories) supplemented with platelet-derived growth factor-BB (PDGF-BB; Peprotech, Cranbury, NJ, USA) for 12 and 24 h at a concentration of 20 ng/mL. The human monocytic THP-1 cell line was purchased from (Fenghui Biotechnology, Changsha, China) and cultured in RPMI-1640 with 10% FBS. The human umbilical vein endothelial cell (HUVEC) line was purchased from ScienCell Research Laboratories and grown in endothelial cell medium (ECM; ScienCell Research Laboratories) with 5% FBS. All cell lines were used at passages 4–7 for all experiments.

**Ligation injury and adeno-associated virus infection of mouse common carotid artery**

ELAVL1^flox/flox^ mice were generated by flanking exons 2 through 5 of the *Elavl1* gene with loxP sites[1]. To ablate ELAVL1 specifically in the smooth muscle (ELAVL1^SMKO^), ELAVL1^flox/flox^ mice were crossed with transgenic mice expressing Cre under the control of the α-smooth muscle actin (α-SMA/*Acta2*) promoter[2]. Both ELAVL1^flox/flox^ and α-SMA-Cre lines were on a C57BL/6J background. All animals were housed under 12 h day/night cycles at 25 °C.

A total of 24 10-week-old male wild-type (WT) or ELAVL1^SMKO^ mice were used. Smooth muscle cell-specific adeno-associated virus (AAV9-SM22a) was manufactured by Genechem Co., Ltd (Shanghai, China). AAV9 (2 × 10^11^ vector genomes (vg)/mouse) was injected via the tail vein one week before surgery in the AAV9-SM22a-Circ_280 group. The control group received the empty AAV9 vector (E.vector). The mouse surgery protocol was performed as previously described[3, 4]. Isoflurane (4%) blended with oxygen (100%, 0.5–1.5 L/min) was used as the inhalable anesthetic during the induction phase. Isoflurane (2%) blended with oxygen (100%, 0.4–0.8 L/min) was used as the inhalable anesthetic during the maintenance phase. The plantar reflex, corneal reflex, and muscle tension of mice were monitored to avoid hyper-anesthesia. Surgery was performed on the left common carotid artery (CCA) of C57BL/6 and ELAVL1^SMKO^ mice; the left CCA was separated from the surrounding connective tissue and completely ligated. The right CCA served as the sham control. Twenty-one days post-ligation surgery, mice were fully anesthetized with sodium pentobarbital (80 mg/kg, i.p.) and perfused with 0.9% saline. Mice were then euthanized by cervical dislocation, and CCAs were harvested. All animal experiments were approved by the Animal Care Committee of Shandong University and were carried out in accordance with the Animal Management Rules of the Chinese Ministry of Health (Document No. 55, 2001). All processes were in accordance with the guidelines of the NIH Guide for the Care and Use of Laboratory Animals.

**Plasmid constructs**

The has_circ_0000280 overexpression plasmid was generated via the insertion of the hsa-circ-0000280 sequence into the PLCDH-ciR vector (Genpharma, Shanghai, China). Transfection of the cells was carried out using Lipofectamine 3000 (Invitrogen, Carlsbad, CA, USA) according to the manufacturer’s instructions and verified by quantitative PCR (qPCR).

**RNase R treatment**

RNA (2 µg) was incubated at 37 °C for 10 min in the presence or absence of 3 U/μg RNase R (Geneseed Biotech Co., Ltd., Guangzhou, China), according to the manufacturer’s instructions, and analyzed by PCR (described below) and agarose gel electrophoresis.

**Agarose gel electrophoresis**

PCR products were separated via 2% agarose gel electrophoresis in TAE buffer with a 500 or 1 000 bp DNA ladder (Takara Bio, Shiga, Japan). The gels were photographed using ImageQuant LAS 4000 (GE Healthcare, Little Chalfont, UK).

**Small interfering RNA (siRNA) transfection**

siRNAs were transfected into VSMCs using Lipofectamine® RNAiMAX (Invitrogen) according to the manufacturer’s instructions. The siRNA for hsa_circ_0000280 was designed according to the sequences of the junction point. The following siRNA sequences were developed and synthesized by Shanghai GenePharma Co., Ltd. (Shanghai, China): ELAVL1-homo Forward 5’-GAACGAAUUUGAUCGUCAATT, and Reverse 5’-UUGACGAUCAAAUUCGUUCTT; CDKN1A-homo Forward 5’-GAUGGAACUUCGACUUUGUTT, and Reverse 5’-ACAAAGUCGAAGUUCCAUCTT; hsa_circ_0000280 Forward 5’-UUGAAGGAUUGAAAGGGAGTT, Reverse 5’-CTCCCUUUCAAUCCUUCAATT.

**Cell Counting Kit-8 (CCK-8) assay**

One day after transfection, cells were harvested and seeded into 96-well plates at a concentration of 3 × 10^3^/well. Zero, 6, 24, and 48 h after, the medium was replaced with 100 μL fresh medium containing 10 μL CCK8 solution (5 mg/mL; Solarbio, Beijing, China) followed by incubation for 1 h at 37 °C and 5% CO_2_. The absorbance was then read at 450 nm in a Spectra Max i3x Multifunctional microplate reader (Molecular Devices, San Jose, CA, USA).

**Migration assay**

Cells were cultured in 6-well plates up to 100% confluency. The cell layer was then scratched using a 20 μL pipette tip. Cells were rinsed well with PBS and observed using a Nikon Ti-E optical microscope (Nikon, Tokyo, Japan). The Image-Pro Plus 6.0 software (Media Cybernetics, Silver Spring, MD, USA) was used for image analysis.

**Cell cycle analysis**

Effects on the cell cycle were investigated using a previously described protocol[5]. In brief, cells (1 × 10^6^) were detached from the plate, rinsed twice in PBS, fixed in 70% ethanol, and stored at 4 °C before overnight DNA staining with 2.5 μg/mL propidium iodide (PI; Sigma-Aldrich, St. Louis, MO, USA) in the presence of 12.5 μg/mL RNase A (Sigma-Aldrich). The cell numbers at each cell cycle phase were then evaluated using the Guava easyCyte HT flow cytometry system (Millipore, Billerica, MA, USA).

**Nuclear and cytoplasmic fractionation**

Cytoplasmic and nuclear fractions were obtained using the Minute™ Cytoplasmic and Nuclear Extraction Kit (Invent Biotechnologies, Plymouth, MN) according to the manufacturer’s instructions.

**RNA isolation and qPCR**

Total RNA from tissue specimens and cells was isolated using the TRIzol reagent (Life Technologies, Carlsbad, CA, USA). To measure the amount of circRNA and mRNA, cDNA was prepared using the Primescript RT Master Mix (Takara Bio, Shiga, Japan) and qPCR was carried out using the TB Green Premix EX Taq (Takara Bio) on the CFX96 system (Bio-Rad Laboratories, Hercules, CA, USA): one 5 s cycle at 95 °C and 40 cycles of 5 s at 95 °C, and 30 s at 60 °C. The levels of circRNA and mRNA expression were normalized to those of β-actin (*ACTB)* using the 2^−ΔΔCt^ approach. The primer sequences used in this study are disclosed in Table S2. The average cycle threshold for genes was evaluated from a minimum of three separate measurements.

**Western blot analysis**

Proteins were extracted from HASMCs using the commercially available RIPA lysis kit (Solarbio, Beijing, China). The extracted proteins (normalized amounts per sample) were separated by SDS-PAGE and transferred onto PVDF membranes. After blocking in 3% BSA in TBST, the membranes were incubated with primary antibodies against ELAVL1 (12582S, 1:1,000, Cell Signaling Technology, Danvers, MA, USA), CDKN1A (2947S, 1:1,000, Cell Signaling Technology), CDK2 (ab32147, 1:1,000, Abcam, Cambridge, UK), CCND2 (3741S, 1:1,000, Cell Signaling Technology), CCNE1 (20808S, 1:1,000, Cell Signaling Technology), β-tubulin (2128S, 1:1,000, Cell Signaling Technology), GAPDH (5174S, 1:1,000, Cell Signaling Technology), or lamin B (12987-1-AP, 1:1,000, PTG, Wuhan, China) overnight at 4 °C. Following incubation with the respective secondary antibodies (ab6721, 1: 5,000, Abcam), proteins were visualized using the ImageQuant LAS 4,000. Protein signal intensity was analyzed using the Image-Pro Plus 6.0 software.

**Fluorescence in situ hybridization (FISH)**

Sample sections were incubated with proteinase K (20 µg/mL) at 37 °C for 25 min followed by one washing step in pure water and three 5 min washes in PBS (pH 7.4) with gentle shaking on a rocker device. The pre-hybridization solution was added to each section, followed by incubation for 1 h at 37 °C. For the hybridization process, the pre-hybridization solution was first removed before adding the hsa_circ_0000280 probe hybridization solution at 1.5 μM. Sections were incubated in a humidified chamber for hybridization overnight at 37 °C. Following the removal of the hybridization solution, the sections were washed in 2X SSC for 10 min at 37 °C, 1X SSC twice for 5 min each at 37 °C, and 0.5X SSC for 10 min at room temperature. Formamide washing was used for the non-specific hybrids. Finally, images were acquired using a positive fluorescence microscope. The following hsa-circ-0000280 probe (for circular junction points) was used: 5ʹ-CY3-CCTCTTTTTCCTCCCTTTCAATCCTTCAA-CY3-3ʹ.

**RNA immunoprecipitation assay (RIP) and sequencing**

High-throughput sequencing was conducted by Cloud-Seq Biotech (Shanghai, China). The HASMC specimens were lysed in ice-cold lysis buffer, and RIP was performed as previously described[6]. Anti-ELAVL1 antibodies were obtained from Cell Signaling Technology. The RIP assays were conducted using the Magna RIP RNA-Binding Protein Immunoprecipitation Kit (Millipore, Billerica, MA, USA) according to the manufacturer’s instructions.

**RNA pull-down assay**

The T7 transcriptional template was created using the hsa_circ_0000280 sequence with a T7 promoter, and the NheI, and KpnI restriction sites (GenePharma, Shanghai, China). In vitro transcription was performed using the MEGAscript® Kit (Life Technologies, Carlsbad, CA, USA). Briefly, the template and the other components were combined and incubated at 37 °C for 3 h according to the manufacturer’s instructions, followed by RNA purification with the RNeasy MinElute Cleanup kit (Qiagen, Hilden, Germany). Of note, HASMCs were harvested once they reached 100% confluency and lysed using the Pierce IP Lysis Buffer (Thermo Fisher Scientific, Waltham, MA, USA). The template RNA was tagged with the Pierce RNA 3'-Desthiobiotinylation Kit (Thermo Fisher Scientific). The pull-down assay was conducted using the Pierce™ Magnetic RNA–Protein Pull-Down Kit (Thermo Fisher Scientific). The eluate was amplified using RT-PCR and separated using TAE buffer and 2% agarose gel electrophoresis. The template details are provided in Table S3.

**Histological evaluation**

CCAs were harvested within 21 days of injury. The cross-sectional regions of the milieu and intima were evaluated after H&E staining in a blinded manner by single investigator using the Image-Pro Plus 6.0 software (Media Cybernetics). Neointima formation was defined as the ratio of the intimal region to the medial region (I/M).

**Immunohistochemistry (IHC) staining**

IHC staining was carried out as previously described[7]. Briefly, paraffin sections were dewaxed, and rehydrated; subsequently antigen retrieval was performed followed by blocking and incubation with antibodies against α-SMA (1:1,000, Servicebio, Wuhan, China) and CDKN1A (1:400, Servicebio) overnight in a moist chamber at 4 °C. The sections were then rinsed three times in PBST, incubated with an HRP-conjugated secondary antibody (1:200, Servicebio) at room temperature for 15 min, rinsed three times in PBST, and stained with hematoxylin and DAB. Finally, the prepared sections were dehydrated and covered with coverslips.

**Supplementary Tables**

**Table S1. Patient information**

| **Variables, mean ± SD or n (percentage)** | **Control, n = 30** | **CHD, n = 70** | **p-value** |
| --- | --- | --- | --- |
| Stenosis of the left main coronary trunk | 16.67 ± 17.29 | 79.21 ± 17.40 | <0.0001 artery (%) |
| TC (mmol/L) | 4.148 ± 0.17 | 4.040 ± 0.13 | ns |
| LDL-C (mmol/l) | 2.456 ± 0.12 | 2.597 ± 0.12 | ns |
| HDL-C (mmol/L) | 1.303 ± 0.06 | 1.198 ± 0.03 | ns |
| Age (years) | 57.57 ± 1.39 | 60.99 ± 1.18 | ns |
| Gender (M/F) | 16 (53)/14 (47) | 49 (70)/21 (30) | ns |
| Diabetes (yes/no) | 4 (13)/26 (87) | 16 (23)/54 (77) | ns |
| Smoking history (yes/no) | 10 (33)/20 (67) | 37 (53)/33 (47) | ns |
| Family history of CHD (yes/no) | 4 (13)/26 (87) | 19 (27)/51 (73) | ns |
| Alcohol intake history (yes/no) | 9 (30)/21 (70) | 26 (37)/44 (63) | ns |
| Other underlying diseases | No | No | ns |
| Pharmacological therapy | Aspirin and clopidogrel | Aspirin and clopidogrel | ns |
| CHD: coronary heart disease; HDL-C: high-density lipoprotein cholesterol; LDL-C: low-density lipoprotein cholesterol; ns: no significance; TC: total cholesterol; SD: standard deviation. | | | |

**Table S2. Primer sequences**

| **Primer** | **Sequence (5ʹ→3ʹ)** | **bp** | **OD** |
| --- | --- | --- | --- |
| hsa_circ_0008731-180 F | AGTTTCCACAGCTGACTGGAG | 21 | 2 |
| hsa_circ_0008731-180 R | AAAAGGAGACTCGACCACTGC | 21 | 2 |
| hsa_circ_0019079-133 F | ATGGCAGTGAAACACCCTGG | 20 | 2 |
| hsa_circ_0019079-133 R | AGTGTCATTCGCATGTCTTCA | 21 | 2 |
| hsa_circ_0001801-128 F | AGCATTGAAACTTCAACCAGGA | 22 | 2 |
| hsa_circ_0001801-128 R | TGGCTTCCAATATTGCACTTGA | 22 | 2 |
| hsa_circ_0000847-120 F | TATTCCAGAAACGCCACCTCC | 21 | 2 |
| hsa_circ_0000847-120 R | GCAAGCCACGCTAGGAAAAC | 20 | 2 |
| hsa_circ_0000280-139 F | AGCCCTTCTGTTTGGGATACT | 21 | 2 |
| hsa_circ_0000280-139 R | AGAGGGTCCTGTAGCTTGGT | 20 | 2 |
| hsa_circ_0008143-160 F | AGGTTTGCAGATTCCGCCAG | 20 | 2 |
| hsa_circ_0008143-160 R | CCCTTGGTCGAATTCTTGCC | 20 | 2 |
| hsa_circ_0004440-132 F | CAACGGAACAAGTTCCAGCAA | 21 | 2 |
| hsa_circ_0004440-132 R | CCCGGTACTTCCCTCTGAGTA | 21 | 2 |
| Homo-hur (ELAVL1)-190F | CCATTAAGGTGTCGTATGCTCG | 22 | 2 |
| Homo-hur (ELAVL1)-190R | CGGATAAACGCAACCCCTCT | 20 | 2 |
| Homo-P21(CDKN1A)-112 F | TGTCTTGTACCCTTGTGCCT | 20 | 2 |
| Homo-P21(CDKN1A)-112 R | TGGTAGAAATCTGTCATGCTGGTC | 24 | 2 |
| Homo-ACTA2-120F | ACTGCCTTGGTGTGTGACAA | 20 | 2 |
| Homo-ACTA2-120R | CACCATCACCCCCTGATGTC | 20 | 2 |
| Homo-sm22-71F | TCAGATGGGCAGCAACAGAG | 20 | 2 |
| Homo-sm22-71R | TGATGATCTGCCGAGGTCGT | 20 | 2 |
| Mus-HuR(Elavl1)-72F | TGGGCGAATCATCAACTCCA | 20 | 2 |
| Mus-HuR(Elavl1)-72R | CGGATAAAGGCAACCCCTCT | 20 | 2 |
| Mus-P21(Cdkn1a)-107F | TTGTCGCTGTCTTGCACTCT | 20 | 2 |
| Mus-P21(Cdkn1a)-107R | TAGAAATCTGTCAGGCTGGTCT | 22 | 2 |
| Homo-HPS5-86F | AGTGTCCCTTGATGCTCCAC | 20 | 2 |
| Homo-HPS5-86R | AACCCCAGGAAAGCAAGTGT | 20 | 2 |
| Mus -Cdk2-199F | CACCCTAATATCGTCAAGCTGC | 22 | 2 |
| Mus -Cdk2-199R | GGTGAAGGACACGGTGAGAA | 20 | 2 |
| Homo-CDK2-100F | TGGATGCCTCTGCTCTCACT | 20 | 2 |
| Homo-CDK2-100R | AGGACCCGATGAGAATGGCA | 20 | 2 |
| Homo- CCND2-176F | CACCTGGATGCTGGAGGTCTG | 21 | 2 |
| Homo- CCND2-176R | TCAGCGGGCTGGTCTCTTTG | 20 | 2 |
| Mus-Ccnd2-158F | TACCTCCCGCAGTGTTCCTA | 20 | 2 |
| Mus-Ccnd2-158R | GCCAAGAAACGGTCCAGGTA | 20 | 2 |
| Homo-CCNE1-151F | AGAGGAAGGCAAACGTGACC | 20 | 2 |
| Homo-CCNE1-151R | TTGTCAGGTGTGGGGATCAG | 20 | 2 |
| Mus-Ccne1-158F | CCTTTCAGTCCGCTCCAGAA | 20 | 2 |
| Mus-Ccne1-158R | GGATGAAAGAGCAGGGGTCC | 20 | 2 |

**Table S3. RNA pull down probes**

| **Probe** | **Sequence** |
| --- | --- |
| hsa_circ_0000280sense | GGAGGAAAAAGAGGTAACTAGTCCACCTCCAGAAGAAGACAGGTTCCAGGAGCTTAAAGTAGCAACAGCAGAAGCAATGACCAAGCTACAGGACCCTCTGGTTTTATTTGAATCCGAGTCTCTGAGAATGGTTTTACAGGAGTGGCTTTCACATTTAGAAAAAACATTTGCCATGAAGGACTTTTCAGGTGTTTCAGATACTGACAACTCATCCATGAAATTGAACCAGGATGTGCTATTAGTTAATGAATCAAAAAAGGGAATATTAGATGAAGATAATGAAAAAGAAAAAAGGGACTCTTTAGGCAATGAAGAATCTGTTGATAAAACAGCATGTGAATGTGTAAGGAGTCCAAGGGAGTCTTTGGATGACCTGTTTCAAATATGTTCTCCATGCGCCATTGCAAGTGGTCTTCGGAACGACCTGGCTGAATTGACAACATTATGTTTGGAGTTGAATGTATTGAATTCTAAGATCAAAAGCACCAGTGGACATGTGGACCACACTTTGCAACAGTACTCTCCTGAAATTCTGGCTTGCCAGTTCCTGAAGAAGTACTTTTTTCTCCTGAACTTGAAAAGAGCGAAGGAGAGTATCAAGCTTAGTTACAGTAATAGCCCTTCTGTTTGGGATACTTTTATTGAAGGATTGAAAG |
| hsa_circ_0000280 antisense | CTTTCAATCCTTCAATAAAAGTATCCCAAACAGAAGGGCTATTACTGTAACTAAGCTTGATACTCTCCTTCGCTCTTTTCAAGTTCAGGAGAAAAAAGTACTTCTTCAGGAACTGGCAAGCCAGAATTTCAGGAGAGTACTGTTGCAAAGTGTGGTCCACATGTCCACTGGTGCTTTTGATCTTAGAATTCAATACATTCAACTCCAAACATAATGTTGTCAATTCAGCCAGGTCGTTCCGAAGACCACTTGCAATGGCGCATGGAGAACATATTTGAAACAGGTCATCCAAAGACTCCCTTGGACTCCTTACACATTCACATGCTGTTTTATCAACAGATTCTTCATTGCCTAAAGAGTCCCTTTTTTCTTTTTCATTATCTTCATCTAATATTCCCTTTTTTGATTCATTAACTAATAGCACATCCTGGTTCAATTTCATGGATGAGTTGTCAGTATCTGAAACACCTGAAAAGTCCTTCATGGCAAATGTTTTTTCTAAATGTGAAAGCCACTCCTGTAAAACCATTCTCAGAGACTCGGATTCAAATAAAACCAGAGGGTCCTGTAGCTTGGTCATTGCTTCTGCTGTTGCTACTTTAAGCTCCTGGAACCTGTCTTCTTCTGGAGGTGGACTAGTTACCTCTTTTTCCTCC |

**Table S4. Significantly different circRNAs in control vs CHD samples**

See the independent file: Supplemental_Table_S4_CHD_vs_ctrl.DE.circRNAseq.xlsx

**Table S5. CircRNA sequencing via ELAVL1 (HuR) RIP assay**

See the independent file: Supplemental_Table_S5_HuR-RIP-seq.xlsx

**Table S6. Top seven significantly downregulated ELAVL1 (HuR) binding circRNAs in CHD samples**

| **circBaseID** | **ID** | **CHD_readcount** | **ctrl_readcount** | **log2FoldChange** | **pval** | **padj** | **[HASMC-HUR.RIP] (raw)** | **[HASMC-HUR.IgG] (raw)** | **[HASMC-HUR.RIP] (normalized)** | **[HASMC-HUR.IgG] (normalized)** | **chrom** | **txStart** | **txEnd** | **strand** |
| --- | --- | --- | --- | --- | --- | --- | --- | --- | --- | --- | --- | --- | --- | --- |
| hsa_circ_0001801 | hg38_circ_0127177 | 72.33084 | 202.1528 | -1.4214 | 1.64E-08 | 1.57E-06 | 4 | 0 | 2 | -2.98457 | chr8 | 52773404 | 52773806 | - |
| hsa_circ_0000847 | hg38_circ_0048486 | 7.841888 | 18.60139 | -1.099 | 0.000218 | 0.003125 | 4 | 0 | 2 | -2.98457 | chr18 | 45391429 | 45423180 | - |
| hsa_circ_0000280 | hg38_circ_0008427 | 4.114526 | 9.956336 | -1.0478 | 3.86E-05 | 0.000794 | 4 | 0 | 2 | -2.98457 | chr11 | 18312988 | 18314523 | - |
| hsa_circ_0008731 | hg38_circ_0107537 | 0.452746 | 2.510622 | -1.9122 | 1.49E-06 | 6.07E-05 | 3 | 0 | 1.584963 | -2.98457 | chr5 | 54941616 | 54960691 | - |
| hsa_circ_0019079 | hg38_circ_0005621 | 0.439064 | 0.819734 | -1.682 | 0.008419 | 0.047972 | 3 | 0 | 1.584963 | -2.98457 | chr10 | 91511102 | 91522592 | + |
| hsa_circ_0008143 | hg38_circ_0069015 | 0.851507 | 2.251797 | -1.0421 | 0.005058 | 0.033002 | 3 | 0 | 1.584963 | -2.98457 | chr20 | 34300940 | 34313077 | - |
| hsa_circ_0004440 | hg38_circ_0052612 | 4.165186 | 9.300461 | -1.0063 | 4.71E-05 | 0.000928 | 3 | 0 | 1.584963 | -2.98457 | chr19 | 45781180 | 45783992 | + |

Top seven significantly downregulated ELAVL1 (HuR) binding circRNAs in CHD PBMCs are shown in detail. Differential expression analysis between groups was performed using DESeq2. The adjusted p-value (padj) is the p-value adjusted for multiple testing using the Benjamini–Hochberg method to estimate the false discovery rate. CircRNAs with a padj < 0.05 and fold change ≤ 0.5 were considered differentially expressed.

**Supplementary Figures**

**
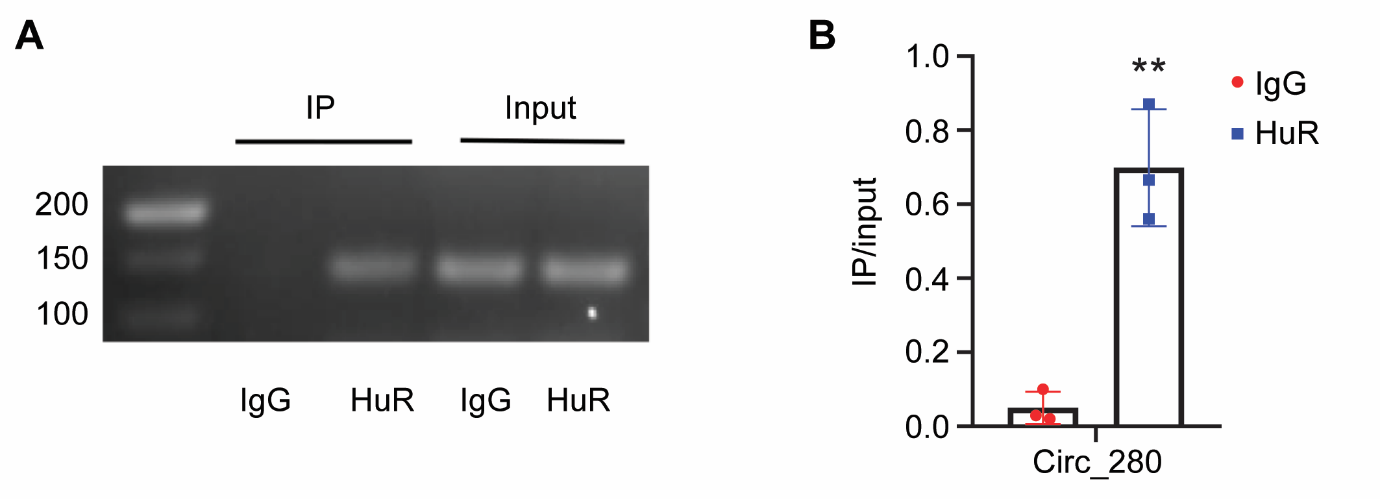
**

**Figure S1.** HuR binds hsa_circ_0000280. **(A)** HuR RIP assay; hsa_circ_0000280 was detected. (**B)** Graphical representation of HuR RIP assay (n = 3, **p < 0.0001).


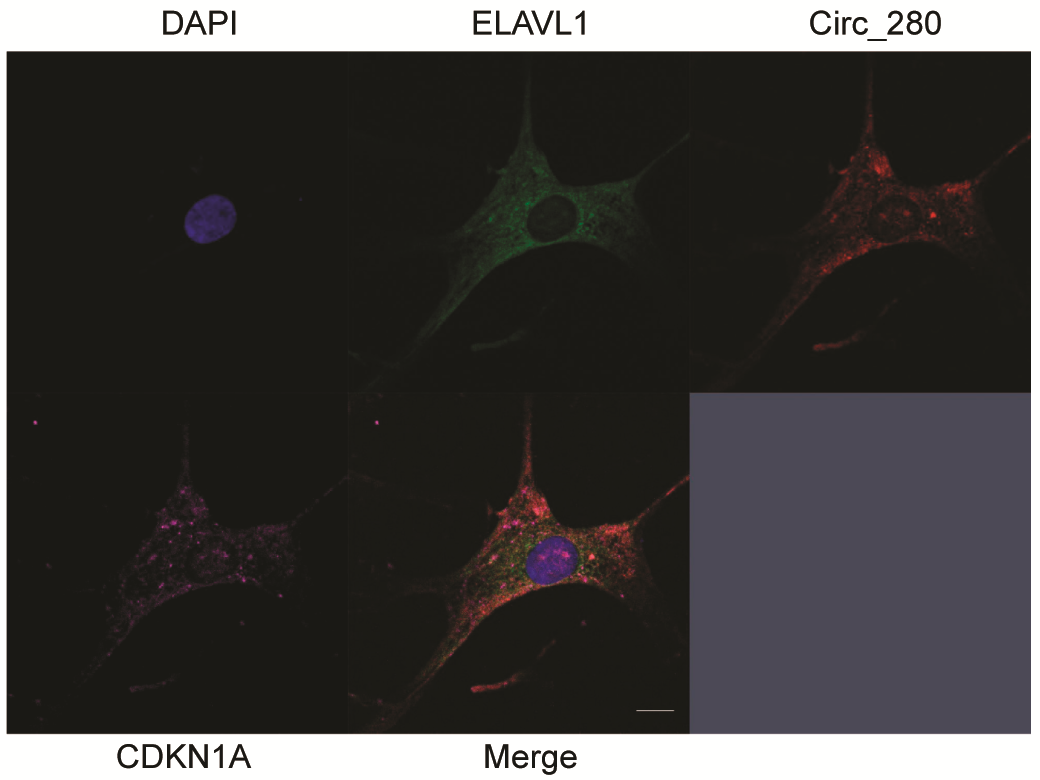

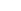

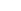


**Figure S2.** Fluorescence in situ hybridization (FISH) assay demonstrating the expression of hsa_circ_0000280, ELAVL1, and CDKN1A. Scale bar, 20 µm.

**
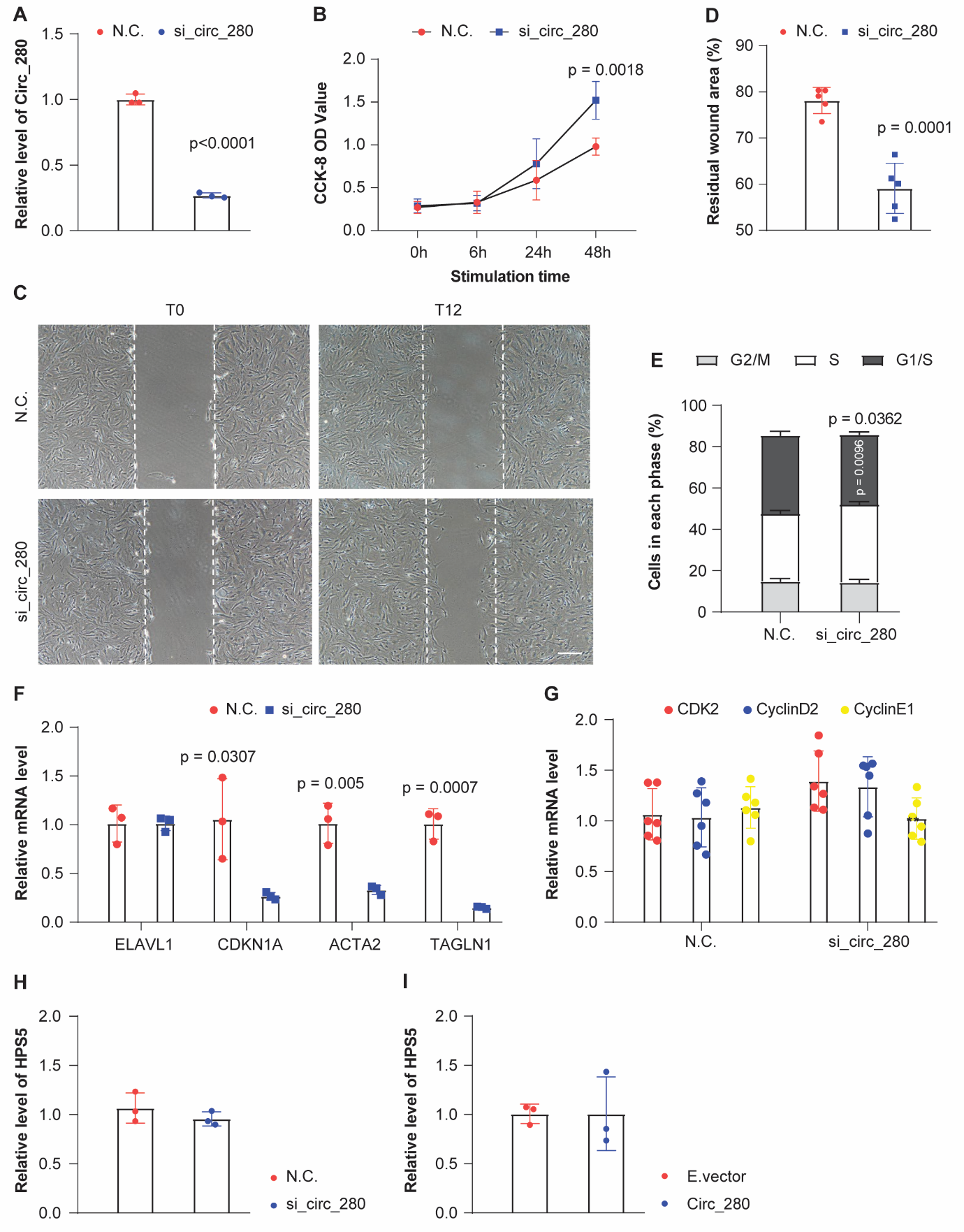
**

**Figure S3.** Biological effects of hsa_circ_0000280 by loss-of-function assay in HASMCs. (**A)** Expression of hsa_circ_0000280 decreased by siRNA (n = 3, p < 0.0001; Student’s *t*-test). (**B)** Measurement of HASMC cell proliferation using CCK8 assay. X-axis: stimulation time of hsa_circ_0000280 siRNA (n = 6, p = 0.0018 vs. 0 h; Student’s *t*-test). (**C)** Wound assay on hsa_circ_0000280-decreased HASMCs. **(D)** Quantification of residual wounded area 12 h post-scratch vs negative controls (N.C.; n = 5, p = 0.0001; Student’s *t*-test). Scale bar, 100 μm. (**E)** Cell cycle assay for hsa_circ_0000280-decreased HASMCs vs N.C. (n = 3, p < 0.05; Student’s *t*-test). **(F)** Expression of HuR, p21, Actin (Acta2), and transgelin (Sm22) in si_circ_280 vs N.C. (n = 3, p < 0.05; Student’s *t*-test). (**G)** Expression level of *CDK2*, CyclinD2 (*CCDN2*), and CyclinE1 (*CCNE1*) by qPCR in hsa_circ_0000280-decreased HASMCs vs N.C. (n = 6, p > 0.05; Student’s *t*-test). (**H, I)** Expression of *HPS5* mRNA following altered hsa_circ_0000280 level (n = 3, p > 0.05; Student’s *t*-test).

**Figure S4.** The expression of circ-280 mutants (n = 6, p>0.05).

**
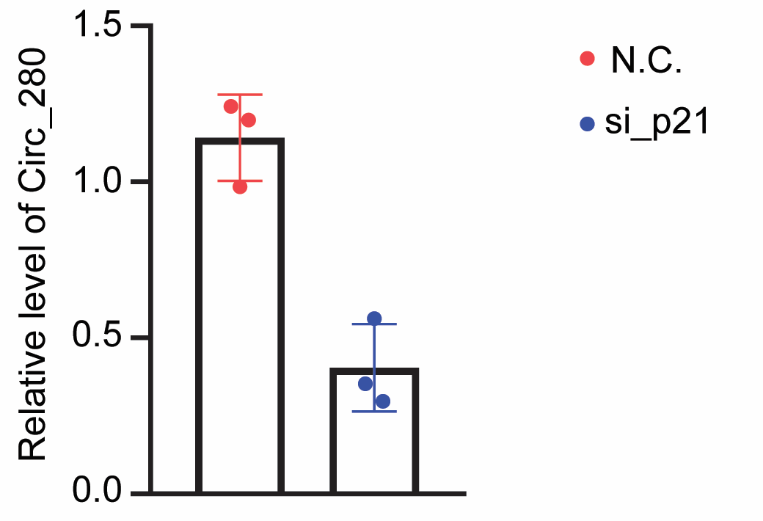
**

**Figure S5.** *P21* mRNA expression inhibited by siRNA (n = 3, p < 0.0001).

**References**

[1] Ghosh M, Aguila HL, Michaud J, Ai Y, Wu MT, Hemmes A, Ristimaki A, Guo C, Furneaux H, Hla T (2009) Essential role of the RNA-binding protein HuR in progenitor cell survival in mice. J Clin Invest 119(12):3530-43.

[2] Wu Z, Yang L, Cai L, Zhang M, Cheng X, Yang X, Xu J (2007) Detection of epithelial to mesenchymal transition in airways of a bleomycin induced pulmonary fibrosis model derived from an alpha-smooth muscle actin-Cre transgenic mouse. Respir Res 8(1):1.

[3] Lee TH, Chen J, Miano JM (2009) Functional characterization of a putative serine carboxypeptidase in vascular smooth muscle cells. Circ Res 105(3):271-8.

[4] Chiang HY, Chu PH, Lee TH (2019) MFG-E8 mediates arterial aging by promoting the proinflammatory phenotype of vascular smooth muscle cells. J Biomed Sci 26(1):61.

[5] Farina FM, Inguscio A, Kunderfranco P, Cortesi A, Elia L, Quintavalle M (2017) MicroRNA-26a/cyclin-dependent kinase 5 axis controls proliferation, apoptosis and in vivo tumor growth of diffuse large B-cell lymphoma cell lines. Cell Death Dis 8(6):e2890.

[6] Zhao J, Sun BK, Erwin JA, Song JJ, Lee JT (2008) Polycomb proteins targeted by a short repeat RNA to the mouse X chromosome. Science 322(5902):750-6.

[7] Wu Y, Zhang Y, Zheng X, Dai F, Lu Y, Dai L, Niu M, Guo H, Li W, Xue X, Bo Y, Guo Y, Qin J, Qin Y, Liu H, Zhang Y, Yang T, Li L, Zhang L, Hou R, Wen S, An C, Li H, Xu W, Gao W (2020) Circular RNA circCORO1C promotes laryngeal squamous cell carcinoma progression by modulating the let-7c-5p/PBX3 axis. Mol Cancer 19(1):99.

**a**
